# Family with sequence similarity 46 member a confers chemo-resistance to ovarian carcinoma via TGF-β/Smad2 signaling

**DOI:** 10.1080/21655979.2022.2064652

**Published:** 2022-04-23

**Authors:** Suiying Liang, Yueyang Liu, Jianhui He, Tian Gao, Lanying Li, Shanyang He

**Affiliations:** a Department of Obstetrics and Gynecology, Guangdong Provincial People’s Hospital & Guangdong Academy of Medical Sciences, Guangzhou, Guangdong, China; b The Second School of Clinical Medicine, Southern Medical University, Guangzhou, Guangdong, China

**Keywords:** FAM46A, chemo-resistance, ovarian cancer, TGF-β signaling pathway

## Abstract

Ovarian cancer is the most lethal malignancy with depressive 5-year survival rate, mainly due to patients with advanced stages experience tumor recurrence and resistance to the current chemotherapeutic agents. Thus, exploring the underlying molecular mechanisms involved in chemo-resistance is crucial for management of treatment to improve therapeutic outcomes. In the current study, we found overexpression of FAM46A in ovarian cancer patients demonstrated an aggressive phenotype and poor outcome. Furthermore, FAM46A overexpression in ovarian cancer cells induces higher CDDP resistance. However, inhibition of FAM46A sensitized ovarian cancer cell lines to CDDP cytotoxicity both in vitro and in vivo. Mechanically, upregulation of FAM46A activated transforming growth factor-β (TGF-β)/Smad signaling and upregulated the levels of nuclear Smad2. Taken together, our results highlight the important oncogenic role of FAM46A in ovarian cancer progression and might provide a potential clinical target for patients with chemo resistant ovarian cancer.

## GRAPHICAL ABSTRACT

**Figure:**
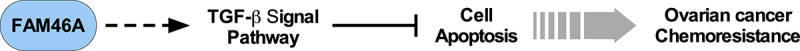

## Introduction

Ovarian cancer (OC) is the sixth most common cancer in women globally and the eighth common cause of cancer death, with overall 5-year survival rate below 45% [1–3]. Although the advances in detection and therapeutics of ovarian cancer, it still represents the most dangerous gynecologic malignancy in women of low and middle income countries [4]. The recommended management for OC is cytoreductive surgery followed by platinum–paclitaxel combination chemotherapy currently; however, more than half of advanced ovarian cancer patients experience chemo-resistance and ultimately bearded tumor relapse and ultimately die of the disease [5,6]. It is an urgent need to clarify the mechanisms underlying chemo-resistance and tumor relapse of ovarian cancer to improve clinical outcomes.

Drug resistance is a complex event which leading to cell tolerance and failure in response to one or multiple clinical pharmaceutical agents [7,8]. Ovarian carcinoma is the most lethal malignancy among women worldwide mainly due to late diagnosis, metastasis within the peritoneal cavity and resistance to chemotherapy [9–11]. The mechanisms of chemo-resistance were classified into two categories, including de novo (intrinsic) and acquired (extrinsic) [12], however, the detailed mechanism of chemo-resistance in ovarian cancer is yet to be understood completely. The TGF-β signaling pathway is a key developmental pathway and it has been reported to play important role in chemo-resistance [13–15]. For instance, it has been reported that overexpression of FAM83A enhances cancer stem cell-like traits and chemoresistance of pancreatic cancer cells by activating TGF-β signaling pathway [16]. Moreover, Park and colleagues reported that TGF-β1 and hypoxia-dependent expression of MKP-1 leads tumor resistance to death receptor-mediated cell death in tumors [17]. The above studies suggest that TGF-β signaling plays an important role in cancer progression and inhibition smad2 signaling may prevent recurrence and chemo-resistance in ovarian cancer. Therefore, the discovery of novel molecules capable of regulating aberrant activation of the TGF-β signaling pathway may facilitate the treatment of chemo-resistant in ovarian cancer.

Family with sequence similarity 46, member A (FAM46A), location in Chromosome 6 open reading frame 37, was originally identified in the diffuse panbronchiolitis critical region of the class I human MHC [18]. It has been reported that deregulation of FAM46A was association with hemoglobinization, ectoderm differentiation, bone abnormalities, and carcinogenesis [19–22]. However, there are still no reports about the biological effects and molecular mechanisms of FAM46A in ovarian cancer chemoresistant.

In this study, we studied the biological effects and molecular mechanisms of FAM46A proteins in ovarian cancer chemoresistance. We hypothesized that overexpression of FAM46A may confers chemo-resistance to ovarian carcinoma by regulating aberrant activation of the TGF-β signaling pathway. And thus highlight the important oncogenic role of FAM46A in ovarian cancer progression and might provide a potential clinical target for patients with chemoresistant ovarian cancer.

## Materials and methods

### Cell culture

The ovarian cancer cell lines SKOV3, A2780 were purchased from The European Collection of Authenticated Cell Cultures (ECACC), were grown in Dulbecco’s modified Eagle’s medium (DMEM, Invitrogen, Carlsbad, CA, USA) supplemented with 10% fetal bovine serum (FBS, Invitrogen), at 37°C in a 5% CO2 atmosphere in a humidified incubator. The CDDP resistant cell line A2780/cis was grown in 10% FBS RPMI 1640 (2 mM Glutamine + 1 µM cisplatin), at 37°C in a 5% CO2 atmosphere in a humidified incubator. All cell lines were authenticated by short tandem repeat (STR) fingerprinting.

### Patient information and tissue specimens

A total of 184 paraffin-embedded and archived ovarian cancer samples were examined in this study. Clinical information on the samples is summarized in Supplementary Table 1. All tums were staged according to the International Federation of Gynecology and Obstetrics standards (FIGO). Ten freshly collected ovarian cancer tissues were frozen and stored in liquid nitrogen until further use. Prior patient consent and approval from the Institutional Research Ethics Committee were obtained for the use of these clinical materials for research purposes.

### Vectors, retroviral infection, and transfection

The human FAM46A gene was PCR-amplified from cDNA and cloned into pMSCV retroviral vector (Clontech, Mountain View, CA). ShRNAs targeting FAM46A were cloned into the pSuper-retro viral vector. Transfection of plasmids was performed using the Lipofectamine 3000 reagent (Invitrogen, Carlsbad, CA) according to the manufacturer’s instruction. Stable cell lines expressing FAM46A and FAM46A shRNA(s) were generated via retroviral infection using HEK293T cells as previously described and selected with 0.5 μg/ml puromycin for 10 days.

### Western blotting (WB) analysis

WB was performed using anti-FAM46A antibody (Abcam, ab163140, 1:500), anti-p-Smad2 (Cell Signaling Technology, #18338, 1:1000), total Smad2 (Abcam, ab40855, 1:2000), anti-β-catenin (Abcam, ab32572, 1:1000), anti-cleaved caspase 3(Abcam, ab32042, 1:200), anti- cleaved PARP (Abcam, ab32064, 1:1000). The blotting membranes were stripped and re-probed with an anti-α-tubulin (Abcam, ab7291, 1:1000) antibody as a protein loading control [23].

### Xenografted tumor model, IHC, and H&E staining

In the intraperitoneal tumor model, the BALB/c nude mice (5–6 weeks, about 20 g) were randomly divided into four groups (n = 5/group). Four groups of mice were inoculated intraperitoneal with 2 × 10^6^ A2780-Vector, A2780- FAM46A, A2780-cis/shRNA-Vector, A2780-cis/ FAM46A -shRNA#1 cells, respectively treated with CDDP (5 mg/kg) every 4 days for 35 days. Tumors were detected by an IVIS imaging system twice a week. Mice was sacrificed in 35 days. Survival was evaluated from the first day of treatment initiation until death and tumors were excised and paraffin-embedded. Apoptotic index was measured by percentage of TUNEL-positive (TUNEL Assay kit(ab66110)) and active caspase 3-positive (Abcam, ab32042, 1:50) cells [24].

### Cytotoxicity assay

The sensitivity to cisplatin of ovarian cancer cells was determined using the MTT assay. Briefly, 2 × 10^3^ cells were seeded onto 96-well plates and incubated at 37°C overnight. Cells were then transfected with different concentrations of cisplatin (0–200 μM). After incubation for 72 hours, 50 μl of the MTT solution (0.15%) was added to each well, and the plates were further incubated for 2 hours. One hundred microliters of DMSO was added to solubilize the MTT formazan product. Absorbance at 540 nm was measured with a Falcon microplate reader (BD-Labware). Dose-response curves were plotted on a semilog scale as the percentage of the control cell number, which was obtained from the sample with no drug exposure. IC50 was determined by the intersection of the cisplatin concentration and the midpoint of the 570-nm reading.

### Apoptosis assay

For evaluation of apoptosis, PE Annexin V Apoptosis Detection Kit I (BD Pharmingen) was used. Briefly, 1 × 10^6^ ovarian cancer cells were plated in 10-cm plates and incubated for 24 hours. Treatment was started with cisplatin (10 μM) for 24 hours. Cell morphology was assessed by phase-contrast microscopy. Then, cells were removed from plate by trypsin-EDTA, washed twice with PBS, and resuspended with binding buffer at 10^6^ cells/ml. FITC Annexin V and propidium iodide were added (each at 5 μl/10^5^ cells). Cells were incubated for 15 minutes at room temperature in the dark. Percentage of apoptosis was analyzed with an EPICS XL flow cytometer (Beckman-Coulter). Each sample was analyzed in triplicate.

### Nuclear and cytoplasmic extraction assay

Nuclear fractions were prepared by using the nuclear extraction kit (Active Motif, Carlsbad, CA). Briefly, after drug treatment, cells were pelleted and lysed by vigorous vortex in hypotonic buffer for 15 min. The samples were then centrifuged at 14,000 × g for 1 min; the supernatant was considered cytoplasmic. Insoluble pellets were further lysed in complete lysis buffer for 30 min, and nuclear extracts (supernatant) were collected after a 10-min centrifugation at 14,000 × g. Both cytoplasmic and nuclear fractions were quantified and subjected to Western blot analysis [25].

### Transient luciferase assay

Cells (1x10^4^) were seeded in triplicate in 48-well plates and allowed to settle for 24 h. For each transfection, one hundred nanograms of luciferase reporter plasmids pGL-3-FAM46A or vector and 5 ng of pRL-TK, expressing Renilla luciferase as an internal control, were transfected into cells using the Lipofectamine 3000 reagent (Invitrogen) according to the manufacturer’s instruction. 48 h after transfection, cells were harvested and Luciferase and renilla signals were measured using the Dual Luciferase Reporter Assay Kit (Promega) according to a protocol provided by the manufacturer. The luciferase activity was normalized by the Renilla luciferase activity of each transfection to normalize the transfection efficiency. Three independent experiments were performed, and the data are presented as mean ± SD [26].

#### Chemical reagents

Cisplatin (Sigma, Saint Louis, MO) were dissolved in PBS with concentration of 50 mM. TGF-β inhibitor were purchased from Santa Cruz Biotechnology (Dallas, TX).

#### Statistical analysis

Statistical tests for data analysis included Fisher’s exact test, log-rank test, Chi-square test, and Student’s 2-tailed t test. Multivariate statistical analysis was performed using a Cox regression model. Statistical analyses were performed using the SPSS 21.0 statistical software package. Data represent mean ± SD. *P* < 0.05 was considered statistically significant.

### Microarray data process and visualization

Microarray data were downloaded from the GEO database: (http://www.ncbi.nlm.nih.gov/geo/).

GSEA was performed using GSEA 2.0.9:(http://www.broadinstitute.org/gsea/).

## Results

In our study, we intended to investigate the biological function and specific regulatory mechanism of FAM46A in ovarian cancer. By performing both i*n vitro* and *in vivo* assay, we hypothesized that overexpression of FAM46A may confers chemo-resistance to ovarian carcinoma by regulating aberrant activation of the TGF-β signaling pathway. And thus highlight the important oncogenic role of FAM46A in ovarian cancer progression and offering novel target genes for the diagnosis and treatment of ovarian cancer chemo-resistance.

### FAM46A is overexpression in chemo-resistance ovarian cancer tissues

**Figure 1.**
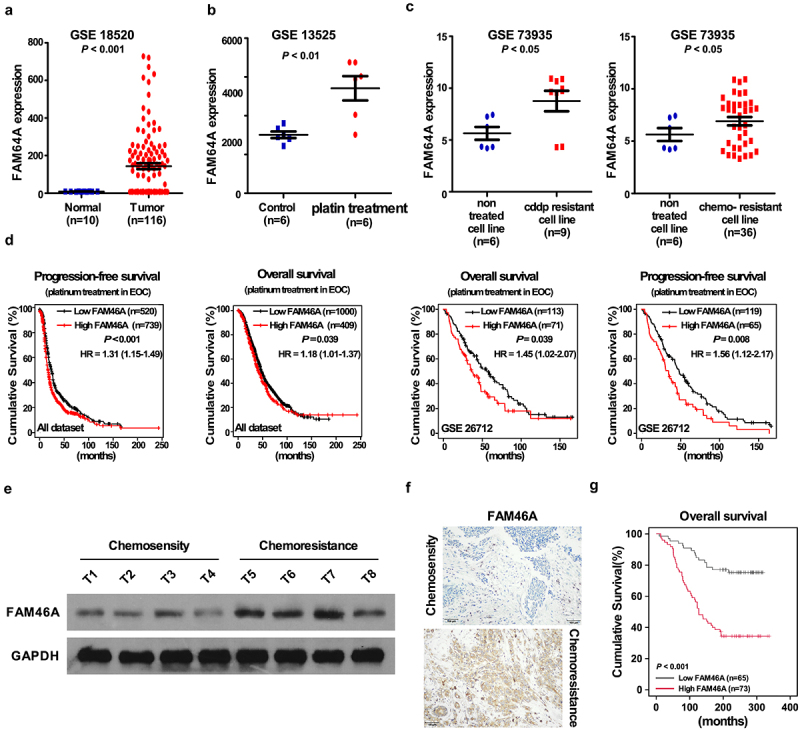
Overexpression of FAM46A correlates with ovarian cancer progression and poor prognosis. (a). Expression profiling of mRNAs showing that FAM46A is upregulated in ovarian cancer tissues (T) compared to normal tissues. (b). Expression profiling of mRNAs showing that FAM46A is upregulated in ovarian cancer tissues with platin treatment compared to control tissues. (c). Expression profiling of mRNAs showing that FAM46A is upregulated in cisplatin resistance ovarian cancer tissues compared to control tissues. (d). Kaplan-Meier analysis of overall or progression-free survival curves from public dataset for ovarian cancer patients with low FAM46A expression or high FAM46A expression. *P < 0.05. (e). Western blotting analysis of FAM46A expression in chemosensity tissues and chemoresistant tissues. (f) IHC staining indicating the FAM46A protein expression in chemosensity tissues and chemoresistant tissues. (g) The Kaplan-Meier survival curves compare ovarian cancer patients with low and high FAM46A expression levels (n = 184; *P* < 0.05).

By analyzing the multiple published mRNA expression profiles (GSE 18520, GSE 13525, GSE 73935) obtained from NCBI (https://www.ncbi.nlm.nih.gov/geo/), we found that the level of FAM46A mRNA was not only upregulated in ovarian cancer tissues compared with normal tissues (Figure 1(a)), but also significantly upregulated in ovarian cancer cell lines with platin treatment or CDDP-resistance (Figure 1(b–c)). Further analysis of Kaplan-Meier plotter-Ovarian cancer datasets showed that ovarian cancer patients with higher FAM46A expression had a shorter survival time and an earlier relapse survival time (P < 0.05; Figure 1(d)). Consistently, western blotting analyses revealed that FAM46A was markedly overexpressed in all four chemo-resistance ovarian cancer tissues, compared with chemo-sensity ovarian cancer tissues (Figure 1(e)).

To determine the clinical relevance of FAM46A in ovarian cancer, FAM46A expression was examined in 184 paraffin-embedded, archived ovarian cancer tissues by IHC assay. As showed in Figure 1(f) and Supplementary Table 1–2, FAM46A levels were correlated with the FIGO stage (*P* = 0.001), and differentiation-state (*P* = 0.009) in patients with ovarian cancer. The increased expression of FAM46A was detected in chemo-resistance ovarian cancer tissue samples, but not chemo-sensity ovarian cancer tissue samples (Figure 1(f)). Importantly, statistical analysis showed that ovarian cancer patients with high FAM46A expression had significantly worse overall and disease-free survival than those with low FAM46A expression (Figure 1(g) and Supplementary Table 3–4). These results suggested that FAM46A is overexpression in chemo-resistance ovarian cancer tissues and might has the potential biomarker for disease outcome prodection in ovarian cancer.

### *Upregulation of FAM46A contributes cytotoxicity of ovarian cancer cells* in vitro

**Figure 2.**
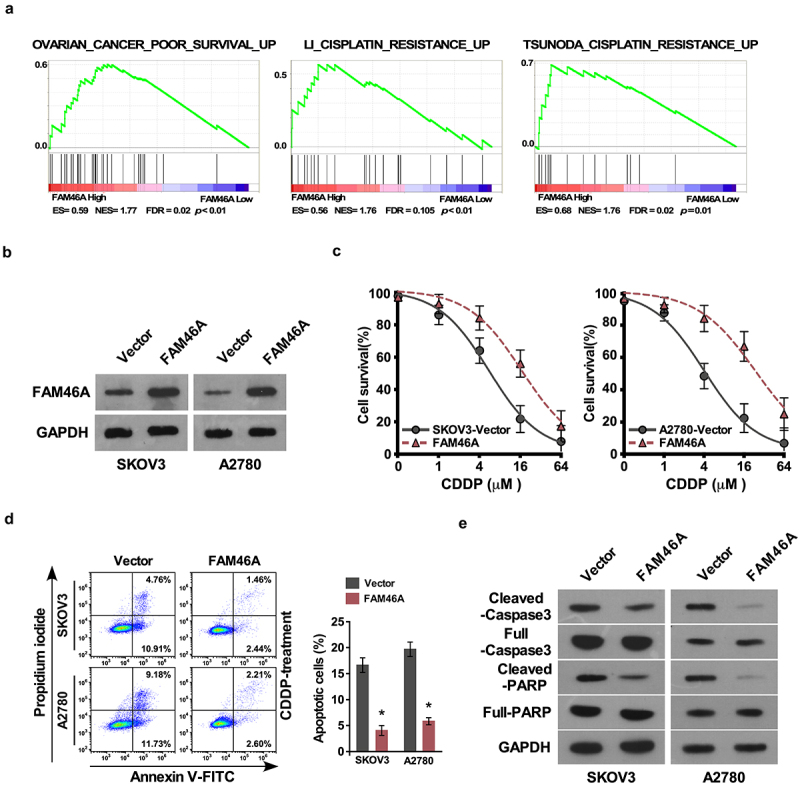
Upregulation of FAM46A conferred ovarian cancer to CDDP resistance *in vitro.* (a). GSEA plot, indicating a significant correlation between the mRNA levels of FAM46A expression in ovarian cancer and the cisplatin resistance gene signatures in TCGA-ovarian cancer datasets. (b) Western blotting analysis of the expression levels of FAM46A proteins in the indicated cells. α-tubulin was used as a loading control. (c) IC50 of CDDP in the indicated cells. (d). Annexin V-FITC and PI staining of the indicated cells treated with cisplatin (10 μM) for 24 h. Each bar represents the mean ± SD of three independent experiments. (e). Western blotting analysis of cleaved caspase3 and PARP in the indicated cells. α-tubulin was used as a loading control.

Upon analyzing TCGA-ovarian cancer dataset via the Gene Set Enrichment Analysis (GSEA) approach, we found a remarkable overlap between high expression profile of FAM46A and cisplatin resistance gene signatures (Figure 2(a)), suggesting that FAM46A might be involved in regulation of ovarian cancer cisplatin resistance. To investigate the chemo-resistance role of FAM46A in ovarian cancer, SKOV3 and A2780 that stably expressed FAM46A cell lines were established (Figure 2(b)). IC50 assay demonstrated that overexpression of FAM46A were resistant to cisplatin than vector-transfected cells (SKOV3:IC50 values were 6.87, and 17.85 μM, respectively; A2780:IC50 values were 4.91, and 21.81 μM, respectively; P < 0.01) (Figure 2(c)). Furthermore, the Annexin V assay show that the percentage of apoptotic cells in FAM46A-overexpression ovarian cancer cells treated with CDDP was much lower compared than that in control cells (Figure 2(d)). The protein level of cleaved caspase 3 and cleaved PARP was significantly decrease in FAM46A overexpression ovarian cancer cells compared with that in control cells (Figure 2(e)). The above results indicated that upregulation of FAM46A is involved in CDDP resistance of ovarian cancer cells.

### *Silencing FAM46A inhibits ovarian cancer CDDP resistance* in vitro

**Figure 3.**
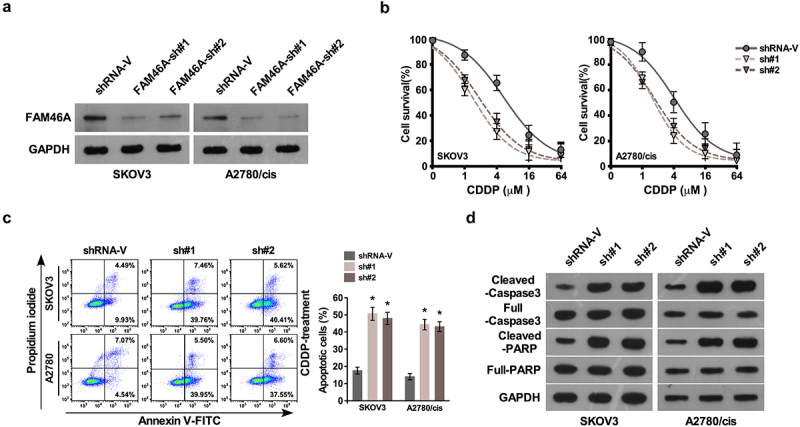
Downregulation of FAM46A sensitized ovarian cancer to CDDP treatment *in vitro.*(a) Western blotting analysis of the expression levels of FAM46A proteins in the indicated cells. α-tubulin was used as a loading control. (b) IC50 of CDDP in the indicated cells. (c). Annexin V-FITC and PI staining of the indicated cells treated with cisplatin (10 μM) for 24 h. Each bar represents the mean ± SD of three independent experiments. (d). Western blotting analysis of cleaved caspase3 and PARP in the indicated cells. GAPDH was used as a loading control.

In agreement with gain of function of FAM46A in ovarian cancer CDDP resistance, silencing FAM46A in SKOV3 and A2780/Cis cell lines significantly decreased the IC50 of CDDP and increase the percentage of apoptotic cells (Figure 3(a–c)). The protein level of cleave at in control cells (Figure 3(d)).

### *Upregulation of FAM46A confers CDDP resistance in ovarian cancer* in vivo

**Figure 4.**
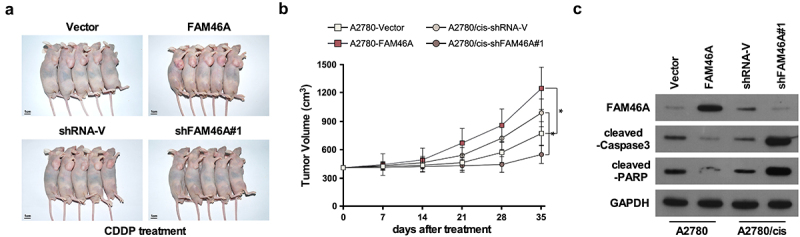
Upregulation of FAM46A confers ovarian cancer to CDDP resistance *in vivo.*(a–b) The luminescence of the intraperitoneal tumor xenografts from different treatment groups at the indicated weeks. (c) Western blotting analysis of the indicated proteins, * *P* < 0.05.

We determined whether deregulation of FAM46A expression was effective in intraperitoneal tumor growth *in vivo*. Nude mice were intraperitoneally inoculated with A2780/Vector and A2780/FAM46A; A2780/cis, A2780/cis/FAM46A shRNA respectively, mouse was treated with CDDP when the treatment with drugs started as soon as the tumor became palpable. As shown in Figure 4(a-b), treatment with FAM46A-shRNA plus cisplatin resulted in a significantly reduction, but overexpression FAM46A resulted in a significantly increase in tumor growth compared with that in the control group. Consistently, analyzing in an *in vivo* intraperitoneal mice model showed that genetically engineered FAM46A conferred great resistance to chemotherapy-induced apoptosis on intraperitoneal growth of A2780 cell, as determined by proportion of active caspase 3 cells compared with that in the control group (Figure 4(c)). However, silencing FAM46A via FAM46A -shRNA enhance the cytotoxic effect of CDDP on ovarian cancer cells, which resulted in remission tumor progression and increased active caspase 3 cells compared with that in the control group (Figure 4(c)). Therefore, these results demonstrated that overexpression of FAM46A contributes to ovarian cancer chemo-resistance *in vivo*.

### Upregulation of FAM46A activates the TGF-β signaling pathways in ovarian cancer

**Figure 5.**
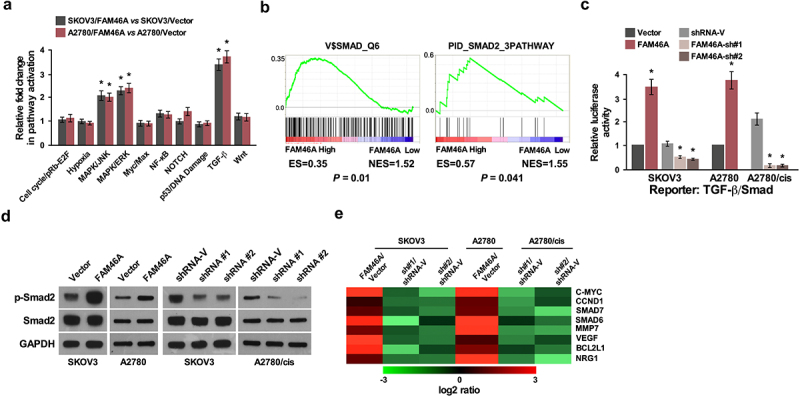
FAM46A up-regulation activates the TGF-β signaling pathway in ovarian cancer. (a) Signal finder reporter arrays showing that overexpression of FAM46A in both SKOV3 and A2780 cells significantly activated NF-κB signaling. Error bars represent the mean ± SD from three independent experiments. (b) GSEA plot, indicating a significant correlation between the mRNA levels of FAM46A expression in ovarian cancer and the TGF-β-activated gene signatures in published datasets. (c) Relative luciferase activities of TGF-β reporter activity in the indicated cells. (d) Western blotting analysis of the expression levels of p-Smad2 proteins in the indicated cells. a-tubulin was used as a loading control. (e) Real-time PCR analysis demonstrating an apparent overlap between TGF-β dependent gene expression and FAM46A–regulated gene expression. The pseudo color represents an intensity scale for FAM46A versus vector or FAM46A siRNA versus control siRNA, calculated by log2 transformation* *P* < 0.05.

To explore the mechanism underlying the effect of FAM46A on promotion of ovarian cancer chemo-resistance traits, Signal finder reporter arrays was performed and revealed that the overexpression of FAM46A in both SKOV3 and A2780 cells resulted in TGF-β pathway activation (Figure 5(a)), suggesting that FAM46A might contribute to modulating TGF-β signaling. Furthermore, GSEA was performed in TCGA dataset in ovarian cancer. We found that FAM46A expression was significantly correlated with activated gene signatures of TGF-β/Smad pathways (Figure 5(b)), suggesting that TGF-β/Smad pathways might contribute to FAM46A mediated chemo-resistance effect on ovarian cancer. As expected, overexpressing FAM46A significantly enhanced, whereas silencing FAM46A reduced, the activities of TGF-β-driven luciferase reporters (Figure 5(c)). Meanwhile, the expression of phosphorylated-Smad2 (p-Smad2) was drastically elevated in FAM46A-transduced cells but decreased in FAM46A-silenced cells (Figure 5(d)). Furthermore, overexpressing FAM46A significantly enhanced, whereas silencing FAM46A reduced the expression of numerous downstream genes of TGF-β pathway (Figure 5(e)). These results suggested that FAM46A plays an important role in activating the TGF-β signaling pathway in ovarian cancer.

### Clinical relevance of FAM46A- induces TGF-β activation in human ovarian cancer

**Figure 6.**
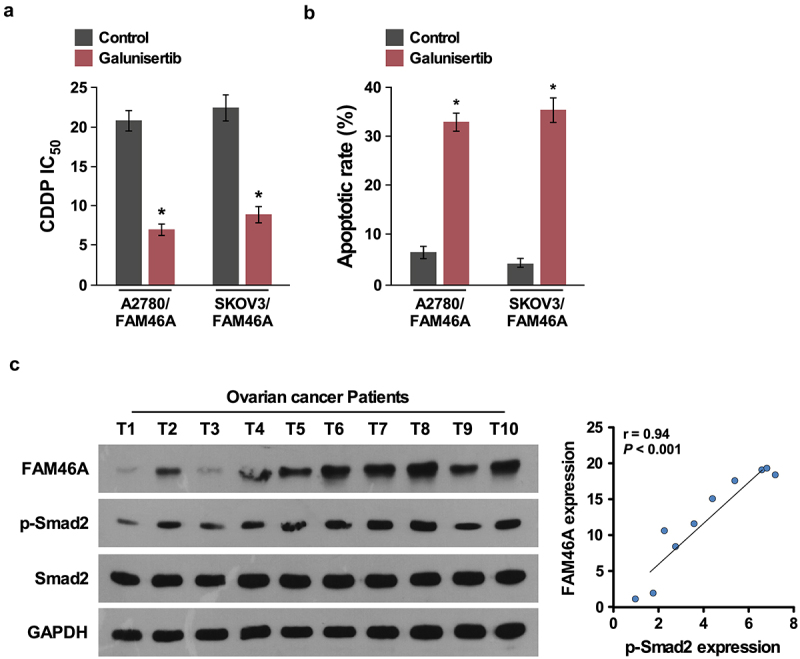
Clinical relevance of FAM46A-induced TGF-β activation in human ovarian cancer. (a). IC50 of CDDP in ovarian cancer cells treated with control or TGF-β inhibitor (100 nM,16 h). (b) Annexin V-FITC and PI staining of the indicated cells treated with control or TGF-β inhibitor(100 nM,16 h). (c).Expression analysis (left) and correlation (right) of FAM46A expression and p-Smad2 (Ser465/467) expression in 10 freshly collected human ovarian cancer tissue samples (t); α-Tubulin was used as loading controls.

Importantly, inhibition of TGF-β/Smad signaling upon TGF-β inhibitor treatment (100 nM,16 h) significantly decreased the IC50 and increased the percentage of apoptotic cells in FAM46A-transduced cells (Figure 6(a)), demonstrating that TGF-β/Smad pathways are functional effectors for chemo-resiatance effect of FAM46A on ovarian cancer. Consistently, FAM46A levels were positively correlated with p-Smad2 expression (r = 0.63; *P* < 0.05) in 10 freshly collected clinical ovarian cancer samples, further suggesting that FAM46A expression was clinically correlated with activities of TGF-β/Smad pathways in ovarian cancer (Figure 6(b)).

## Discussion

In the current study we provided evidence of the potential oncogenic role of FAM46A in ovarian cancer progression and the effect of FAM46A on ovarian cancer chemoresistance. We demonstrated that FAM46A was substantially overexpressed in chemo-resistance ovarian cancer and promoted cancer cell chemoresistance through activation of TGF-β pathways. Hence, our results uncover a novel biological effects and molecular mechanisms of FAM46A proteins in ovarian cancer chemo-resistant and suggest a potential therapeutic target in ovarian cancer.

Numerous studies reported that multiple signaling pathways, such as TGF-β signaling pathways contributed to chemo-resistance of cancer cells. It has been reported that activation of TGF-β pathway promotes tumor heterogeneity in the tumor-initiating cells and leading to drug resistance and tumor recurrence in squamous cell carcinoma [27]. Tripathi et al. also reported that TGF-β-induced alternative splicing of TAK1 promotes epithelial-to-mesenchymal transition (EMT) and drug resistance [28]. Furthermore, Xu and colleagues shown that TGF-β plays a vital role in triple negative breast cancer (TNBC) epirubicin-resistance through regulating stemness, EMT, and apoptosis [29]. However, inhibition of TGF-β pathway by pharmacological inhibitors can decrease the glioma-initiating cells (GICs) population and reduce the capacity of GICs to initiate tumors [30], and suppressed TGF-β signaling can reverses metastasis and chemoresistance of highly malignant NSCLC cells [31], suggesting that TGF-β signaling is a key regulator of chemoresistance and targeting TGF-β signaling make it a challenging target and imply the need for careful therapeutic in cancer.

Our results showed that the overexpression of FAM46A ovarian cancer contributed to cancer cisplatin resistance. However, the mechanism of FAM46A overexpression in ovarian cancer remain unclear. Interestingly, we found that FAM46A exhibited amplification rate of 21.2% in ovarian cancer according to copy number variation analysis of TCGA datasets (https://www.cureline.com/the-cancer-genome-atlas.html), suggesting that the overexpression of FAM46A in ovarian cancer is associated with genomic amplification. Furthermore, analysis of the FAM46A promoter region using the rVISTA program (http://rvista.dcode.org/) predicted three typical NF-κB-responsive elements and two typical STAT3-responsive elements (SRE). It has been previously reported that NF-κB [32] and STAT3 signaling [33] play important roles in progression and development of ovarian cancer. Thus, it would be of great interest to further investigate whether upregulation of FAM46A in ovarian cancer chemo-resistance is attributed to NF-κB and/or STAT3-mediated transcriptional upregulation.

## Conclusion

In summary, our study provides key evidence to support that FAM46A overexpression was involved in ovarian cancer progression and chemoresistance. Expounding the precise role of FAM46A in the pathogenesis of ovarian cancer and molecular mechanism of FAM46A in activation of the TGF-β signaling pathways would increase our knowledge of the biological basis of cancer progression and may also allow the development of new therapeutic strategies against ovarian cancer chemoresistance.

## Supplementary Material

Supplemental Material

## Abbreviations

Cis-dichlorodiammine-platinum,CDDP; Family with sequence similarity 46 member A, FAM46A; transforming growth factor-β,TGF-β); Ovarian cancer,OC; short hairpin RNA, shRNA; Gene Set Enrichment Analysis,GSEA
