# Supplementary material for: Family with sequence similarity 46 member a confers chemo-resistance to ovarian carcinoma via TGF-β/Smad2 signaling
Source: Bioengineered. 2022 Apr 23;13(4):10629–39. doi: 10.1080/21655979.2022.2064652 (PMC9161906; doi:10.1080/21655979.2022.2064652)
Supplement: Supplemental Material [file KBIE_A_2064652_SM2691.zip › supplementary/ethical.pdf]

广东省人民医院医学研究伦理委员会

伦理号：KY-Q-2021-097-02

医学研究伦理委员会审查批件

<table>
<tr><td>项目名称</td><td colspan="6">基于单细胞转录组测序和空间转录组测序的卵巢癌恶性发展的机制和临床价值</td></tr>
<tr><td>项目负责人</td><td colspan="2">何善阳</td><td colspan="2">项目类别</td><td colspan="2">研究者发起的研究，科研项目</td></tr>
<tr><td>审查文件</td><td colspan="6">1.复审申请
2.修改内容对照表
3.卵巢恶性肿瘤标本收集的知情同意书2.0 （版本号：2.0，版本日期：2021年07月19日）
4.正常卵巢标本收集的知情同意书2.0 （版本号：2.0，版本日期：2021年07月19日）</td></tr>
<tr><td>审查类型</td><td colspan="2">复审</td><td colspan="2">评审方式</td><td colspan="2">简易程序审查</td></tr>
<tr><td>评审结果</td><td colspan="6">●批准</td></tr>
<tr><td>审查意见</td><td colspan="6">1、知情同意书建议告知没有补偿。</td></tr>
<tr><td rowspan="2">表决情况</td><td>出席</td><td>人</td><td>投票</td><td>人</td><td>回避</td><td>人</td></tr>
<tr><td colspan="6">批准 人，修改后批准 人，修改后再审 人，暂停或终止研究 人，不批准 人，
备注：简易程序审查</td></tr>
<tr><td>跟踪审查时间</td><td colspan="6">该项目跟踪审查频率为12个月，请在2022-06-25前提交年度定期跟踪审查报告。</td></tr>
<tr><td>主任委员</td><td colspan="2"></td><td colspan="2">日期</td><td colspan="2">2021年07月26日</td></tr>
</table>

注：1.所有涉及人的生物医学研究通过伦理审批后需至“医学研究登记备案信息系统”（网址：http://114.255.48.20）登记备案后方可启动。

2.为充分保证研究对象的权益，本项试验开始后如有研究方案、知情同意书和招募材料等改变，或出现严重不良事件（人体）或其它意外情况，请速报本委员会，本委员会拥有是否终止该试验的权利。

3.研究期限超过一年的项目应将年度进展报告提交伦理委员会跟踪审查。

4.研究结束后请将研究课题结题报告提交伦理委员会审查。

本委员会依据《赫尔辛基宣言》等伦理原则和国家法规的要求操作。
