# Supplementary material for: Family with sequence similarity 46 member a confers chemo-resistance to ovarian carcinoma via TGF-β/Smad2 signaling
Source: Bioengineered. 2022 Apr 23;13(4):10629–39. doi: 10.1080/21655979.2022.2064652 (PMC9161906; doi:10.1080/21655979.2022.2064652)
Supplement: Supplemental Material [file KBIE_A_2064652_SM2691.zip › supplementary/Supplementary information.docx]

**Supplementary table 1. Clinicopathological Characteristics of Studied Patients and Expression of FAM46A in Ovarian Cancer**

| **Characteristics** | **No. of Cases** |
| --- | --- |
| **Age (years)** |  |
| ≤55 | 96 |
| ＞55 | 42 |
| **Lymphatic Transfer** |  |
| Negative | 61 |
| Positive | 30 |
| Unknow | 47 |
| **FIGO Stage** |  |
| I &II | 51 |
| III & IV | 87 |
| **Chemotherapy before surgery** |  |
| Yes | 9 |
| Not | 129 |
| **Differentiation-state** |  |
| Poorly differentiated | 80 |
| Moderately differentiated | 44 |
| Well differentiated | 14 |
| **Status (at follow-up)** |  |
| Alive | 74 |
| Death because of ovarian cancer | 64 |
| Death because of other than ovarian cancer | 0 |
| **FAM46A expression** |  |
| Negative | 3 |
| Positive | 135 |
| Low expression | 65 |
| High expression | 73 |

**Supplementary table 2. Correlation between FAM46A expression and clinicopathological characteristics of ovarian cancer**

| **Characteristics** | | **FAM46A** | | **Chi-square test**  ***P*-value** | **Fisher’s Exact**  **test *P*-value** |
| --- | --- | --- | --- | --- | --- |
|  |  | **Low No. cases** | **High No. cases** |  |  |
| **Age (years)** | ≤55 | 45 | 51 | 0.936 | 0.541 |
|  | ＞55 | 20 | 22 |  |  |
| **FIGO Stage** | I &II | 39 | 12 | 0.000 | 0.000 |
|  | III & IV | 26 | 61 |  |  |
| **Chemotherapy before surgery** | Yes | 3 | 6 | 0.392 | 0.308 |
|  | Not | 62 | 67 |  |  |
| **Differentiation-state** | Poorly | 30 | 50 | 0.009 | 0.009 |
|  | Moderately  Well | 24  11 | 20  3 |  |  |
| **Survive or Mortality** | Yes | 16 | 48 | 0.000 | 0.000 |
|  | No | 49 | 25 |  |  |

**Supplementary table 3. Spearman correlation analysis between FAM46A and clinicopathological characteristics factors**

| **Variables** | **FAM46A Expression Level** | |
| --- | --- | --- |
|  | **Spearman Correlation** | ***P*-Value** |
| **Age** | -0.007 | 0.936 |
| **FIGO Stage** | 0.450 | 0.000 |
| **Chemotherapy before surgery** | 0.073 | 0.396 |
| **Differentiation-state** | 0.251 | 0.003 |
| **Survive or Mortality** | 0.412 | 0.000 |

**Supplementary table 4. Univariate and multivariate analyses of various prognotic parameters in patients with ovarian cancer Cox-regression analysis**

|  | **Univariate analysis** | | | **Multivariate analysis** | | |
| --- | --- | --- | --- | --- | --- | --- |
|  | **No. patients** | ***P*** | **Regression coefficient (SE)** | ***P*** | **Relative risk** | **95% confidence interval** |
| **FIGO Stage** |  | | | | | |
| I &II | 51 | 0.000 | 2.001（0.402） | 0.000 | 5.525 | 2.430-12.557 |
| III & IV | 87 |  |  |  |  |  |
| **Differentiation-state** |  | | | | | |
| Poorly differentiated  Moderately differentiated  Moderately differentiated | 80  44  14 | 0.000 | 0.843(0.241) | 0.007 | 1.978 | 1.205-3.246 |
| **Expression of FAM46A** |  | | | | | |
| Low expression | 65 | 0.000 | 1.331（0.290） | 0.040 | 1.888 | 1.031-3.459 |
| High expression | 73 |  |  |  |  |  |
