# Supplementary figures and images for: Family with sequence similarity 46 member a confers chemo-resistance to ovarian carcinoma via TGF-β/Smad2 signaling
Source: Bioengineered. 2022 Apr 23;13(4):10629–39. doi: 10.1080/21655979.2022.2064652 (PMC9161906; doi:10.1080/21655979.2022.2064652)

Supplementary Figure 1

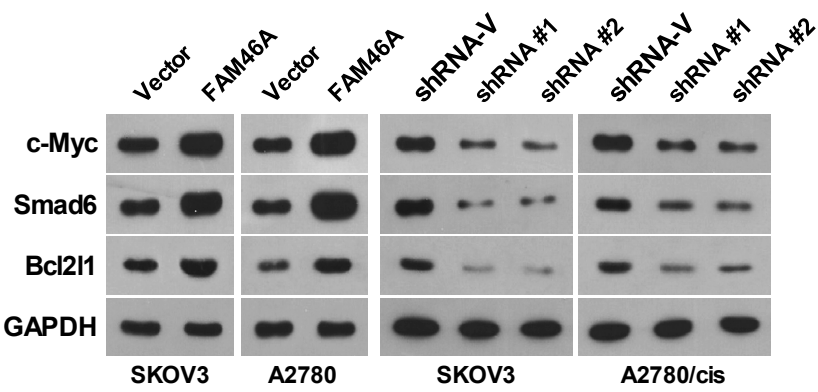

Supplementary Figure 2

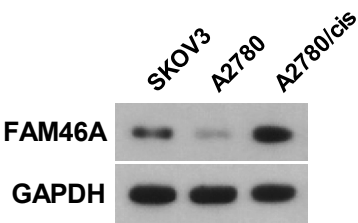

Supplement: Supplemental Material [file KBIE_A_2064652_SM2691.zip › supplementary/supplementary figures.pdf]

Figure 1E

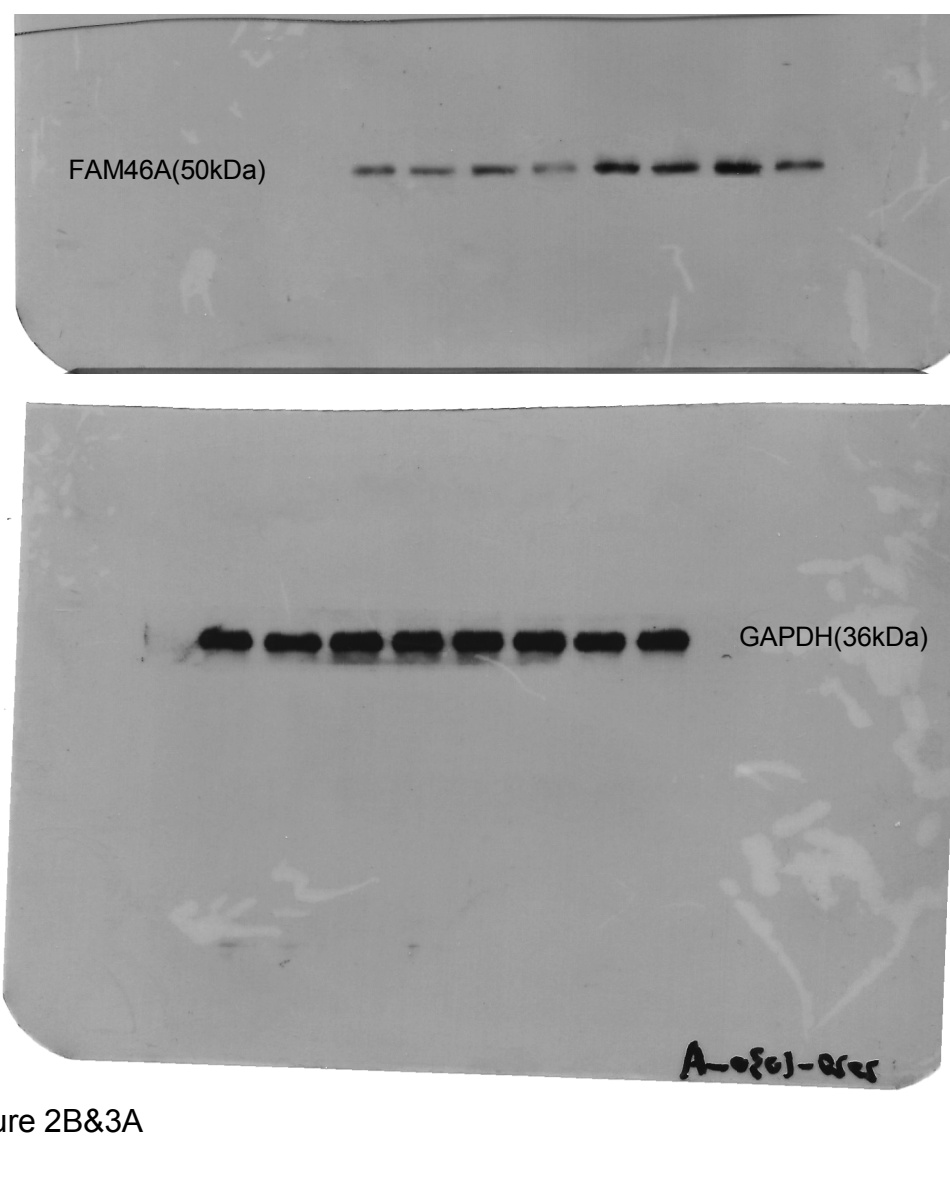

Figure 2B&3A

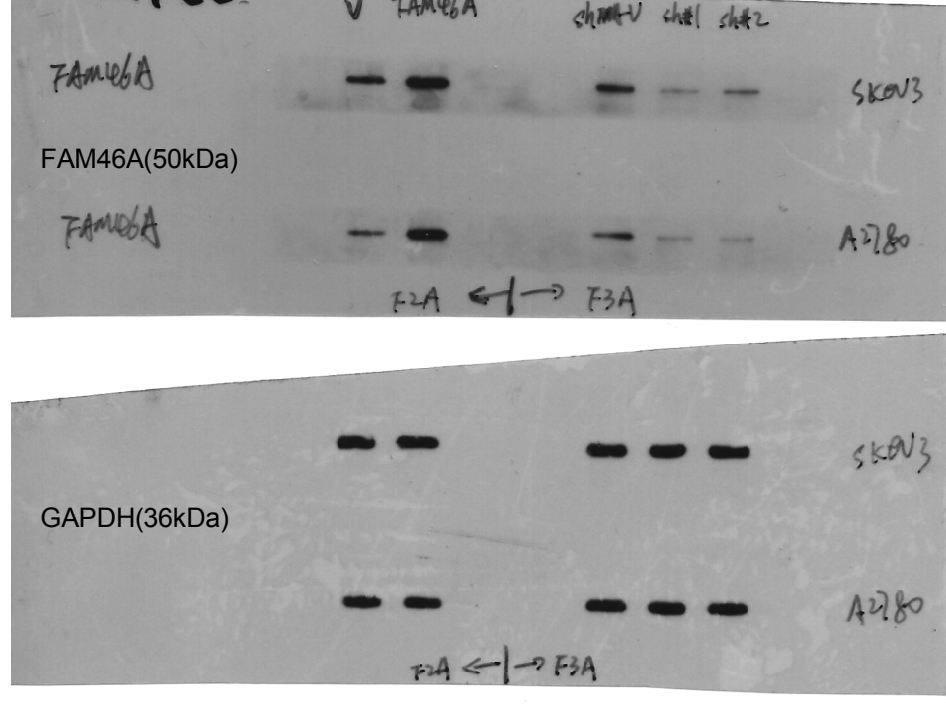

Figure 2E

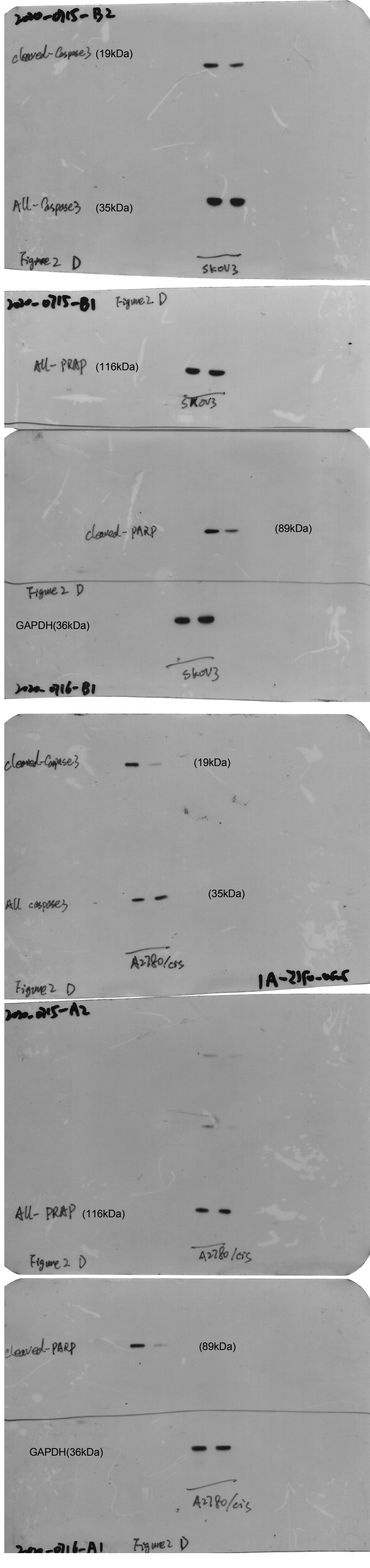

Figure 3D

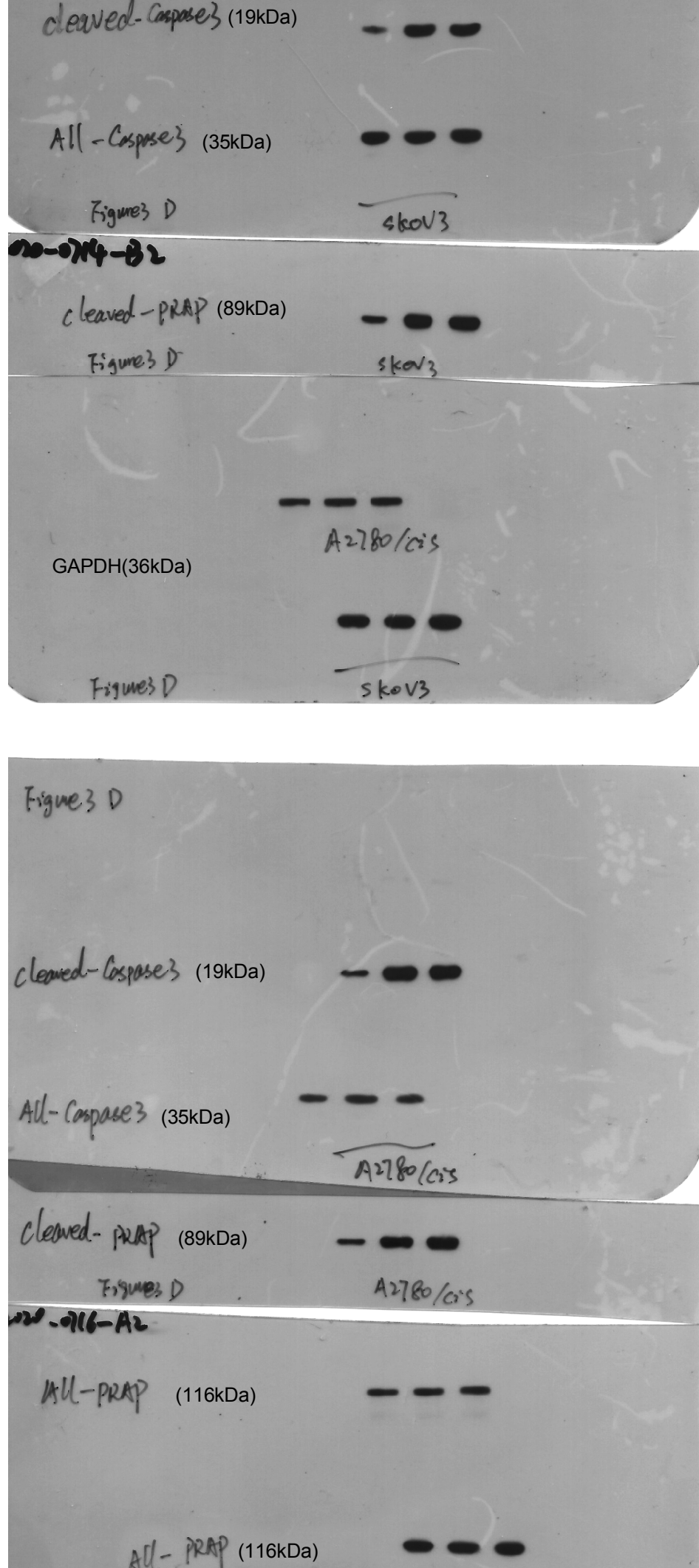

Supplement: Supplemental Material [file KBIE_A_2064652_SM2691.zip › supplementary/westen blot 1.pdf]

Figure 4C

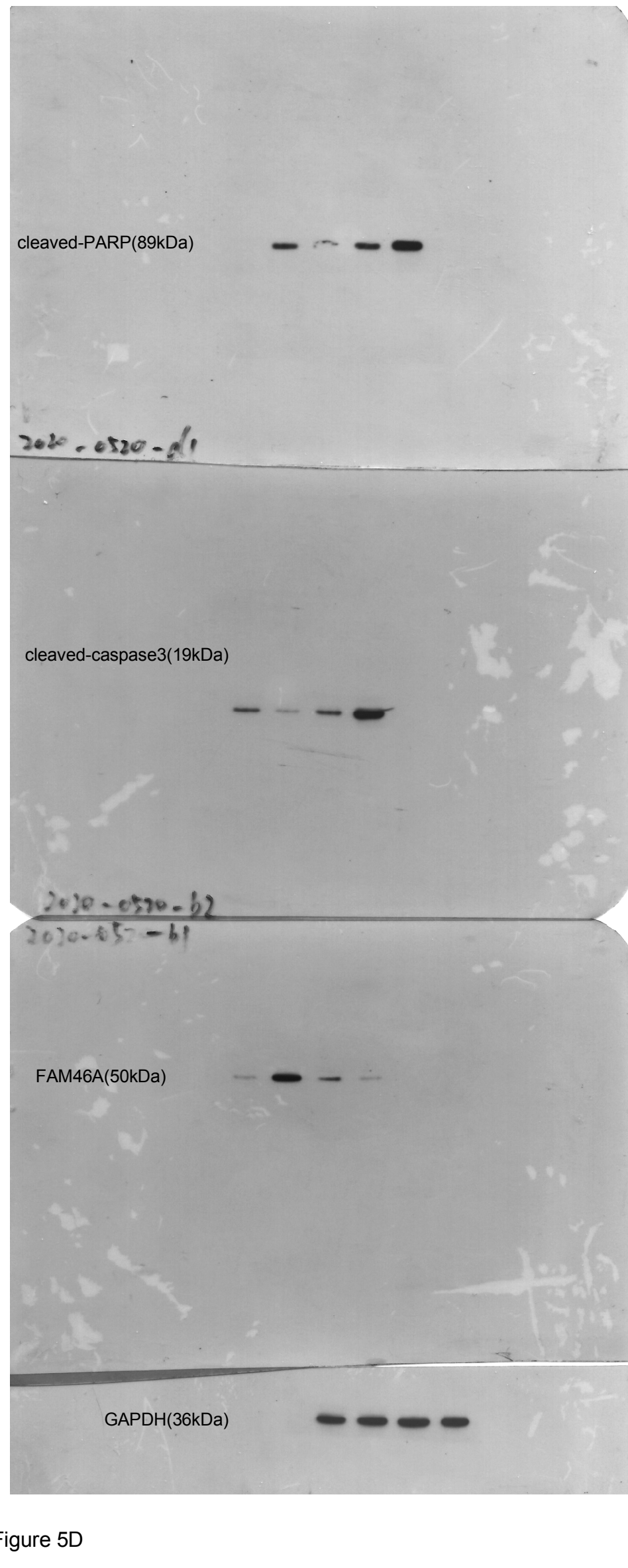

Figure 5D

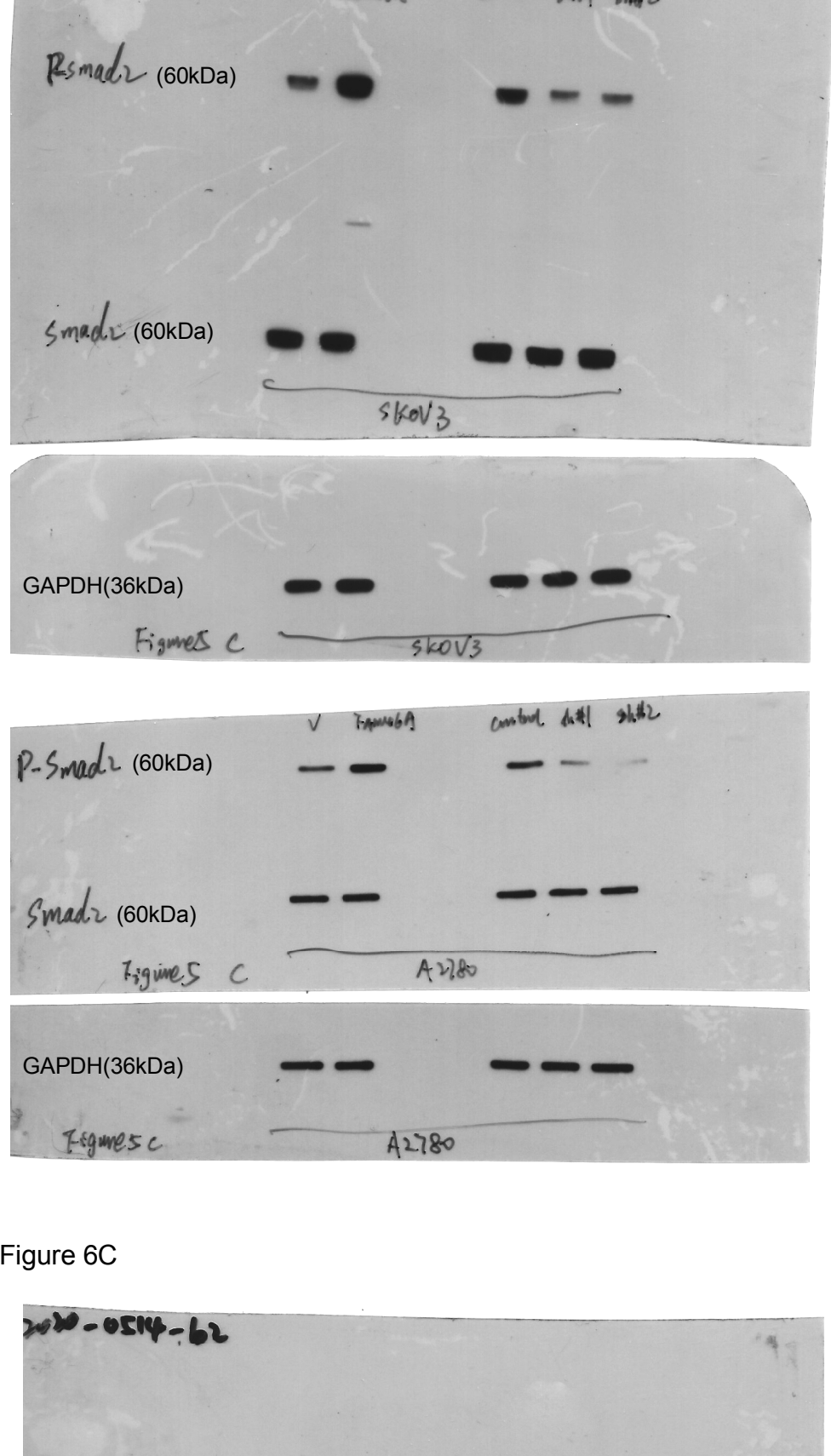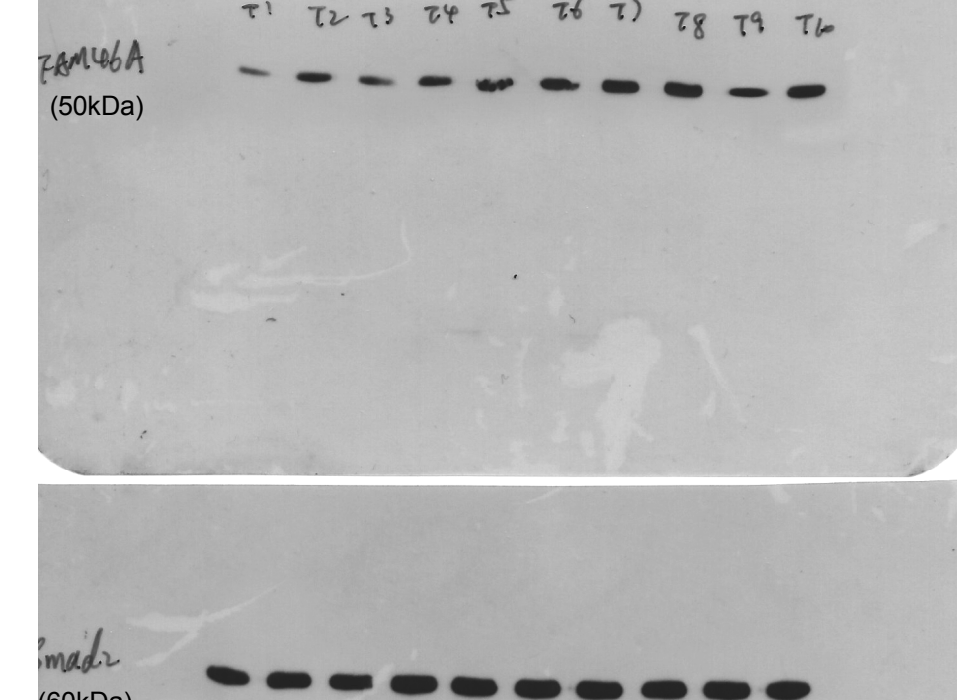

Figure 6C

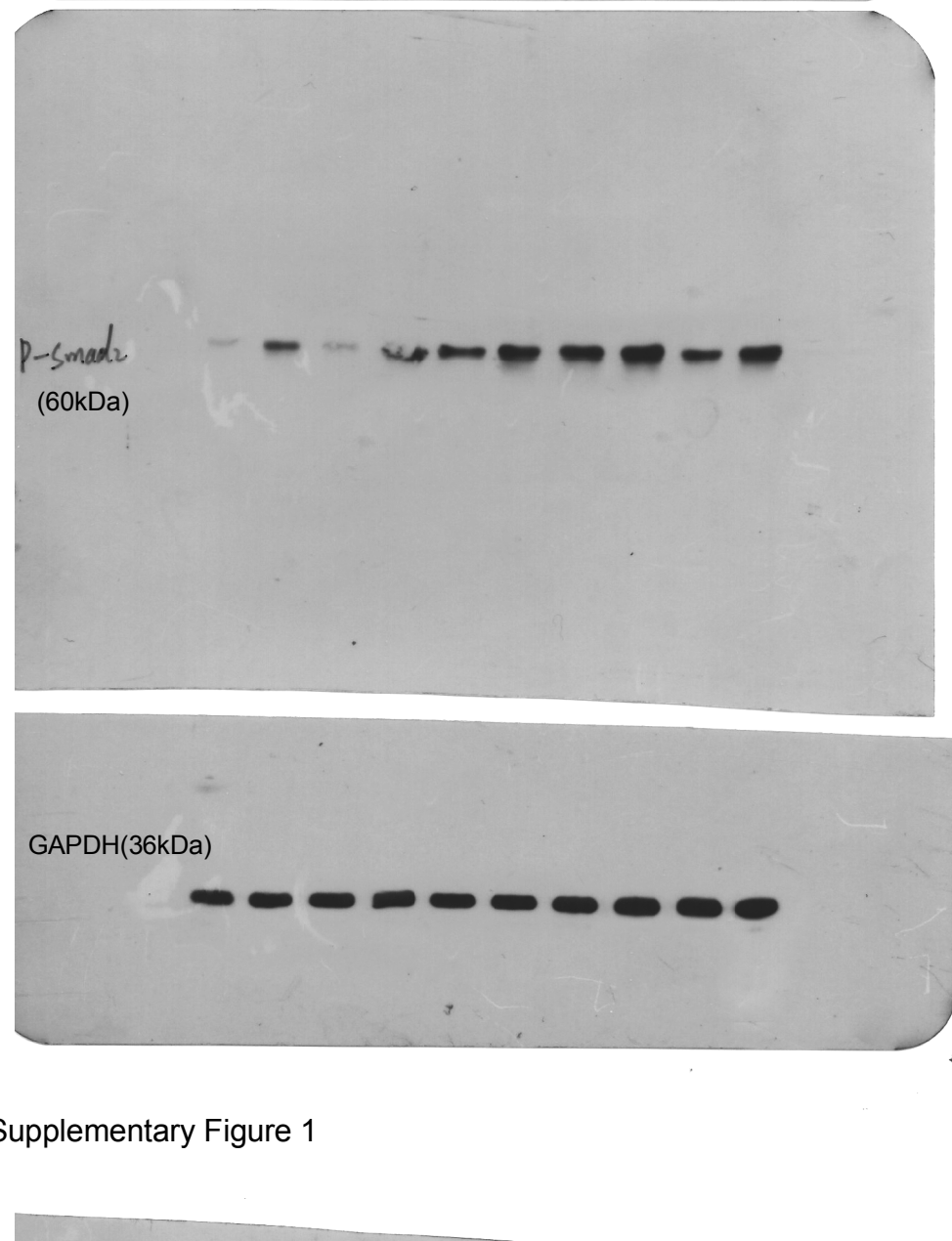

Supplementary Figure 1

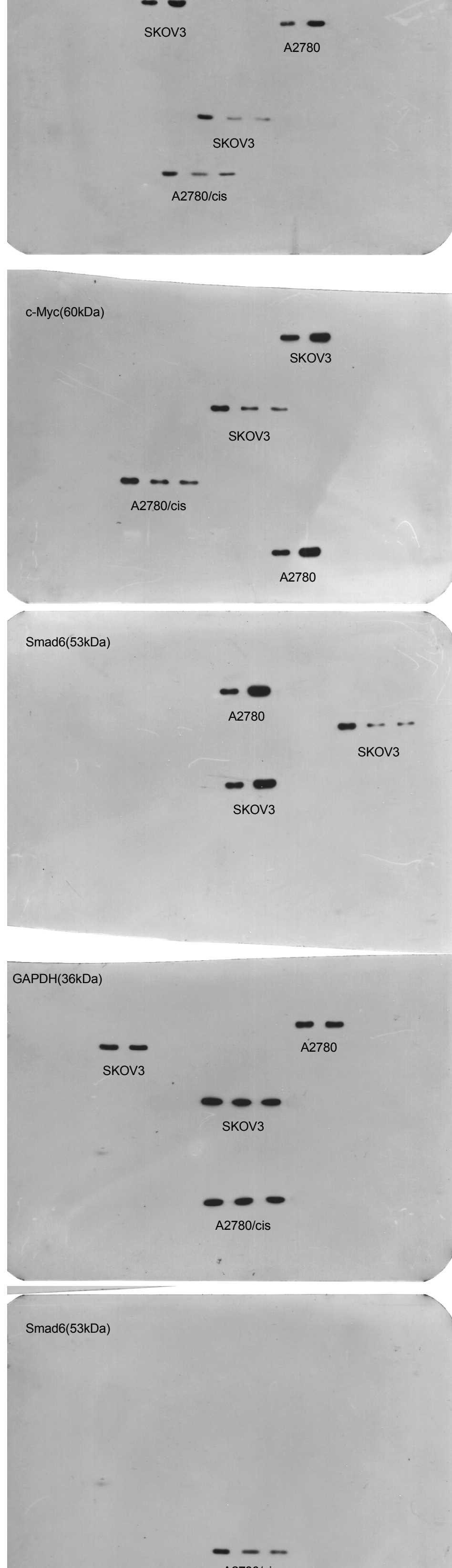

Supplementary Figure 2

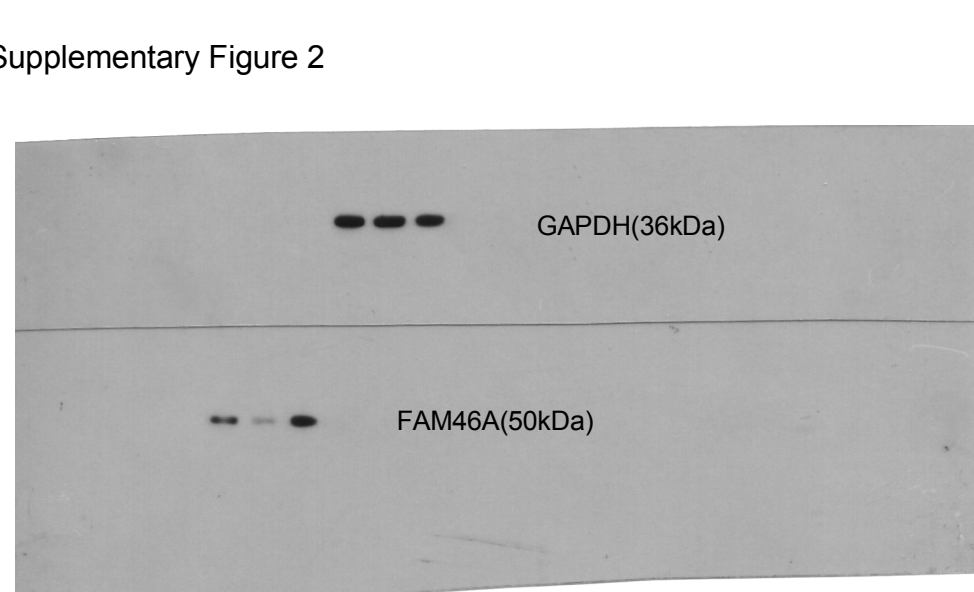

Supplement: Supplemental Material [file KBIE_A_2064652_SM2691.zip › supplementary/western blot 2.pdf]
